# Supplementary material for: Spatial-fluxomics provides a subcellular-compartmentalized view of reductive glutamine metabolism in cancer cells
Source: Nat Commun. 2019 Mar 22;10:1351. doi: 10.1038/s41467-019-09352-1 (PMC6430770; doi:10.1038/s41467-019-09352-1)
Supplement: Supplementary file 1 — Supplementary Information [file 41467_2019_9352_MOESM1_ESM.pdf]

Supplementary Information

Spatial-fluxomics provides a subcellular-compartmentalized view of reductive glutamine metabolism in cancer cells

Lee et al.

Supplementary Figures

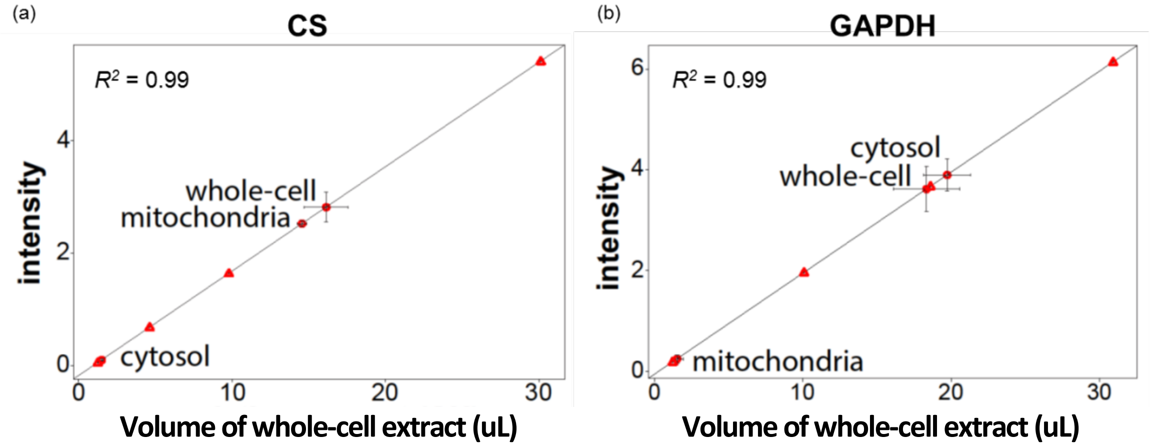

Supplementary Fig. 1: Standard curve for quantitative protein abundance measurement for CS (a) and GAPDH (b) in HeLa cells. Standard curve was generated by loading different volume of the whole-cell sample on the gel (0.8, 5, 10, and 30 µL for CS; 0.8, 5, 20, and 30 µL for GAPDH). The Supplementary Figure shows that the measured abundance of the CS contamination in the cytosolic fraction and GAPDH in the mitochondrial fraction is accurately detected within the linear range. Data are mean ± SD, n = 4 biological replicates.

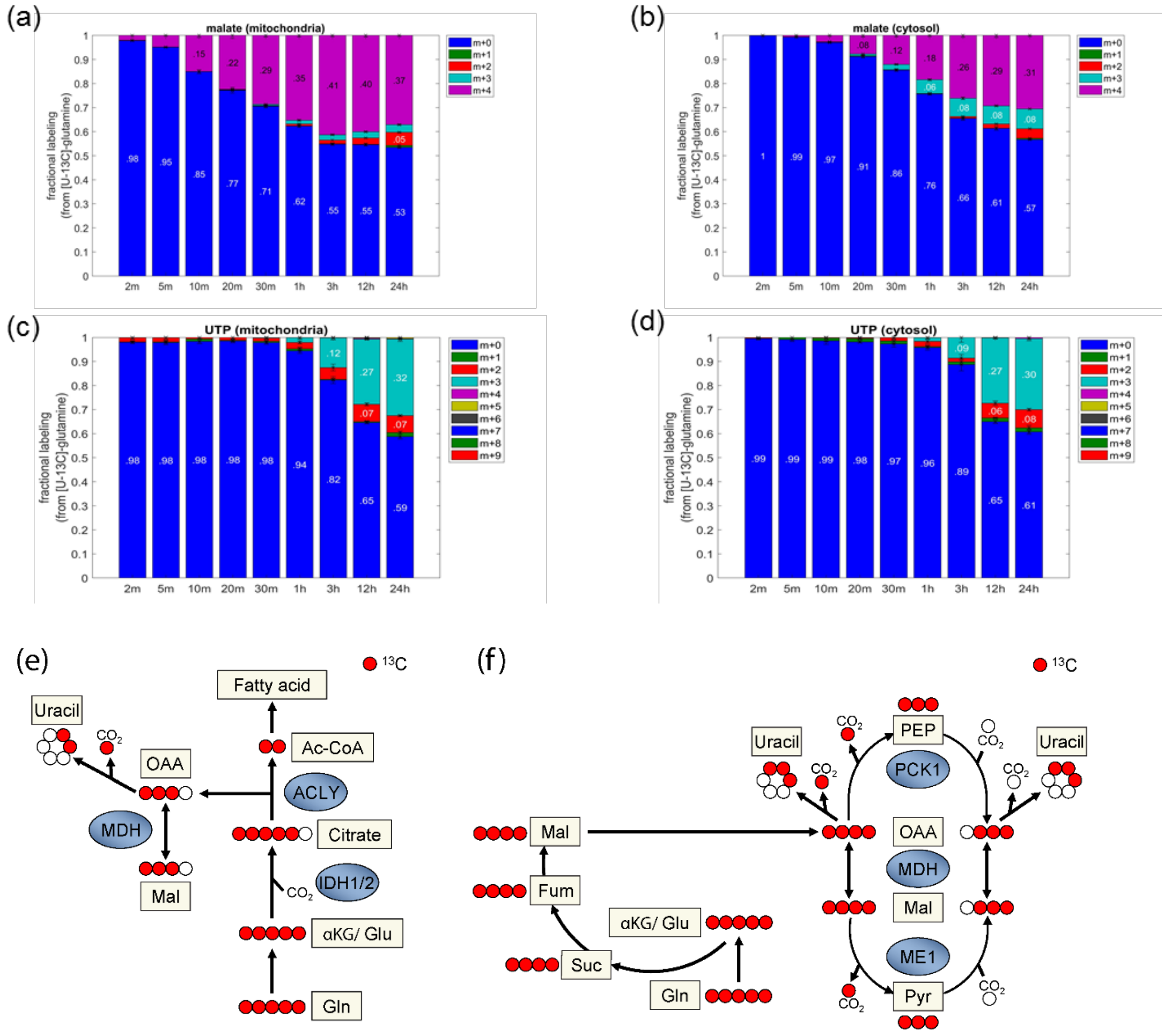

Supplementary Fig. 2: Isotopic labeling kinetics of malate in mitochondria (a) and cytosol (b); and uridine triphosphate (UTP) in mitochondria (c) and cytosol (d) when feeding HeLa cells with [U-^{13}C]-glutamine. (e-f) Alternative routes for synthesizing cytosolic malate m+3: (e) From citrate m+5 via cytosolic ATP citrate lyase (ACLY); and (f) from malate/oxaloacetate m+4 via malic enzyme (ME1) or phosphoenolpyruvate carboxykinase (PCK1), via forward-backward flux, releasing isotopic CO_2 and incorporating non-labeled CO_2. Comparing the isotopic labeling of cytosolic malate and UTP after 24h shows similar fractional labeling of UTP m+3 and malate m+4 of ~30%, suggesting that the entire UTP m+3 pool is produced by malate m+4. with no major contribution of malate m+3 to producing UTP m+3. Hence, malate m+3 produces UTP m+2, via a loss of one isotopic carbon to ^{13}C CO_2 through ME1/PCK1. Data are mean ± SD, n = 3 biological replicates.

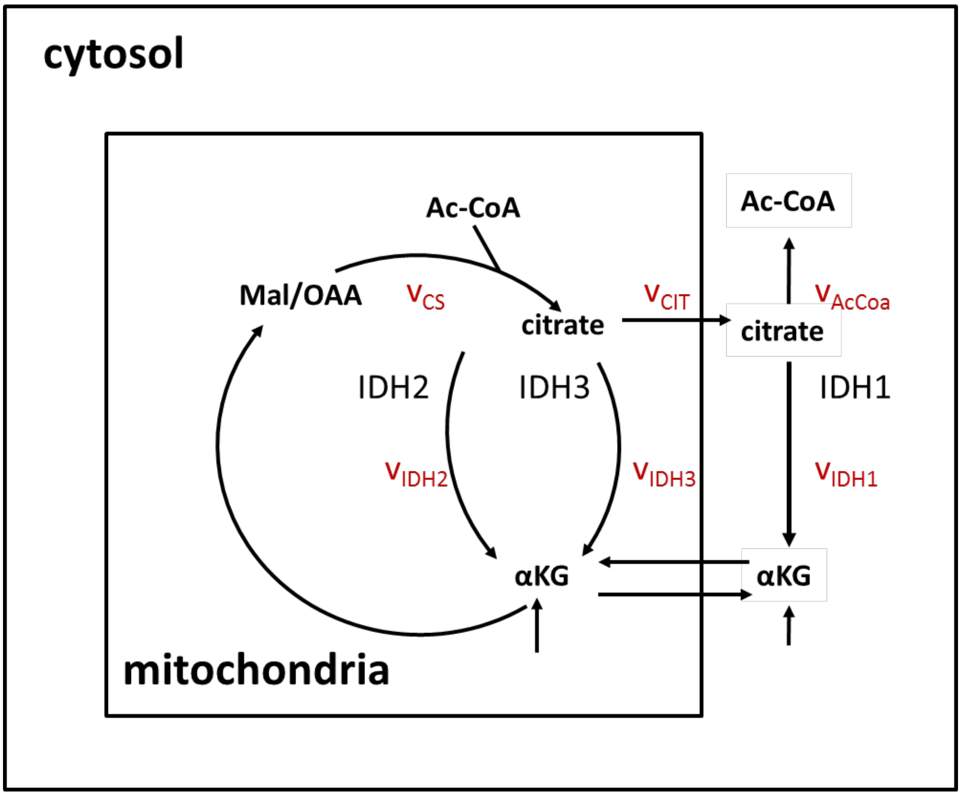

Supplementary Fig. 3: A compartmentalized metabolic network model of citrate metabolism.

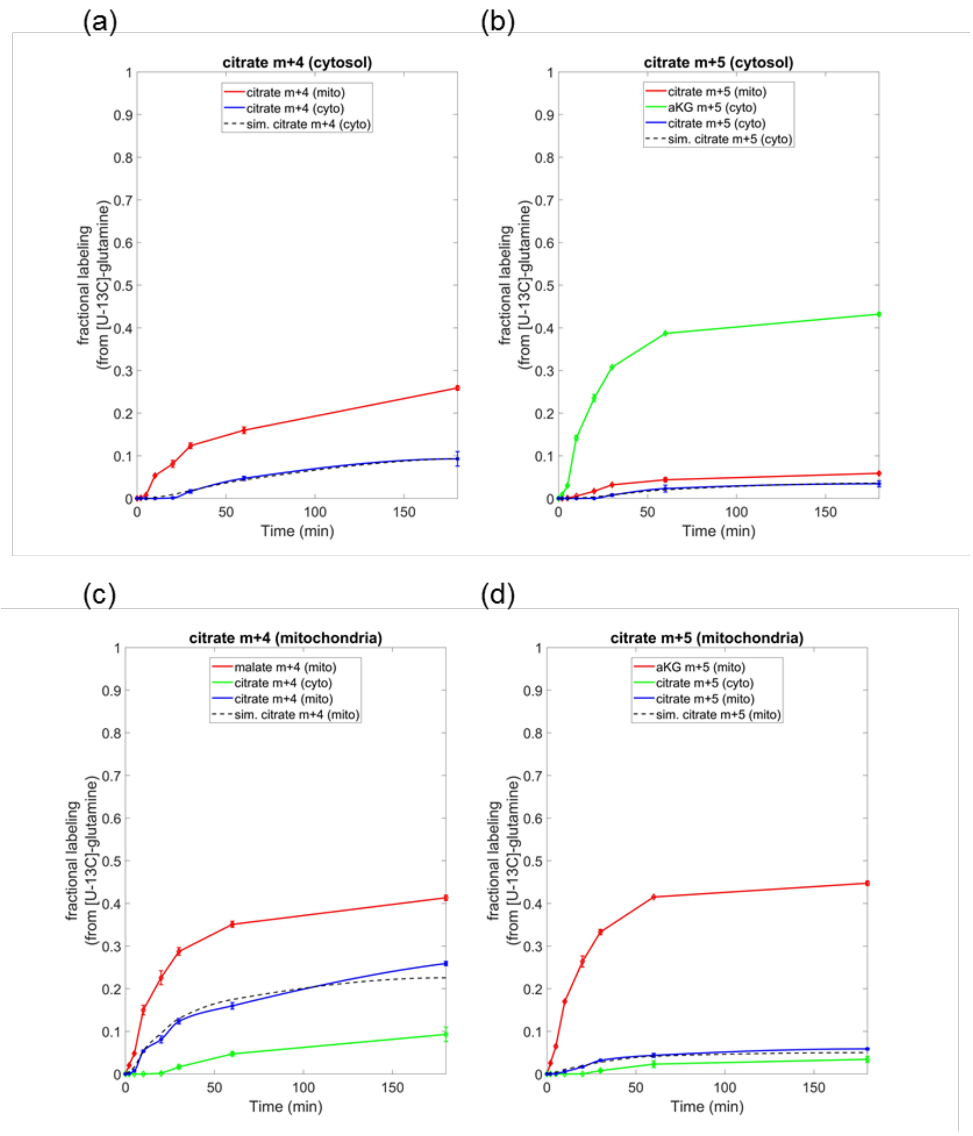

Supplementary Fig. 4: Model simulation of metabolite isotopic labeling kinetics in mitochondria and cytosol in HeLa cells under normoxia. Measured labeling kinetics of cytosolic citrate m+4 (a) and m+5 (b), and mitochondrial citrate m+4 (c) and m+5 (d) after switching cells to [U-^{13}C]-glutamine are in blue, while the corresponding model simulations are in black. The additional curves in each subgraph show the measured labeling kinetics of substrate metabolite pools (citrate, α-ketoglutarate, and malate in mitochondria and cytosol) producing the specific simulated mass-isotopomers of citrate. Data are mean ± SD, n = 3 biological replicates.

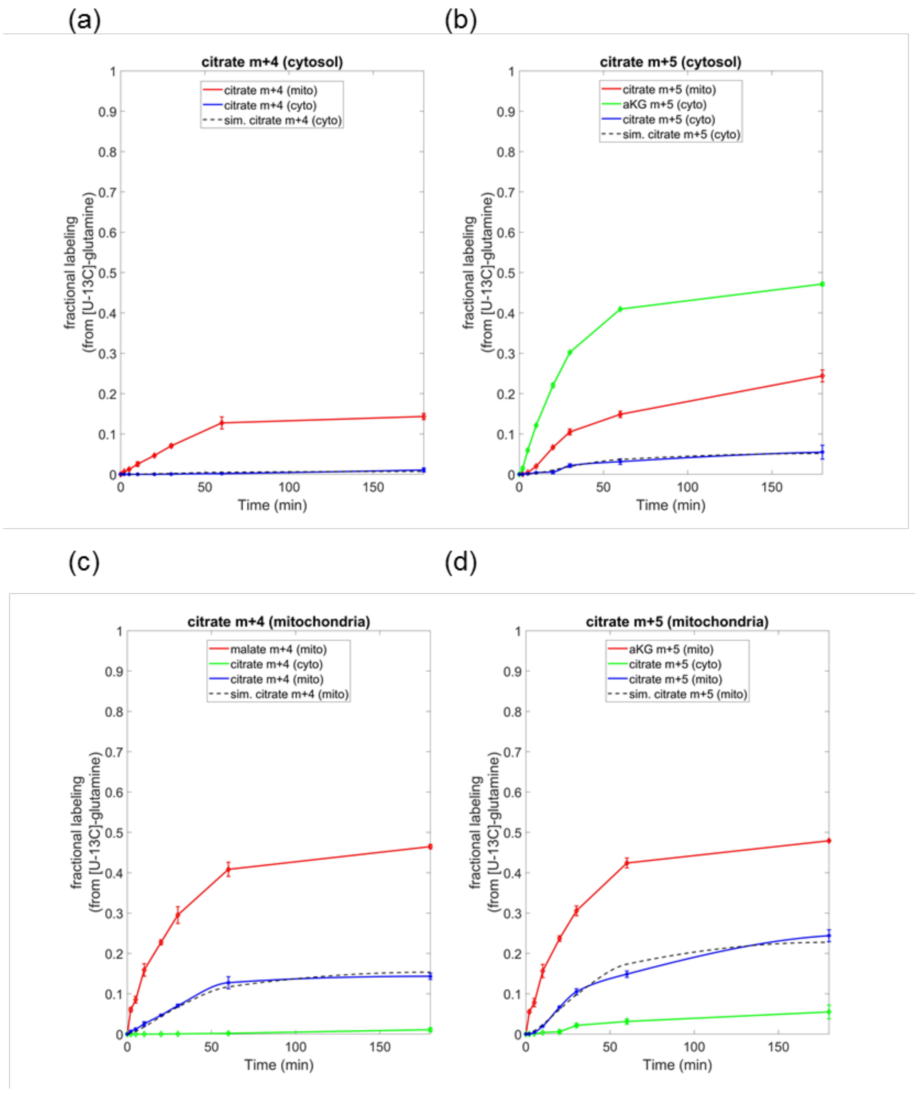

Supplementary Fig. 5: Model simulation of metabolite isotopic labeling kinetics in mitochondria and cytosol in HeLa cells under hypoxia. Measured labeling kinetics of cytosolic citrate m+4 (a) and m+5 (b), and mitochondrial citrate m+4 (c) and m+5 (d) after switching cells to [U-^{13}C]-glutamine are in blue, while the corresponding model simulations are in black. The additional curves in each subgraph show the measured labeling kinetics of substrate metabolite pools (citrate, α-ketoglutarate, and malate in mitochondria and cytosol) producing the specific simulated mass-isotopomers of citrate. Data are mean ± SD, n = 3 biological replicates.

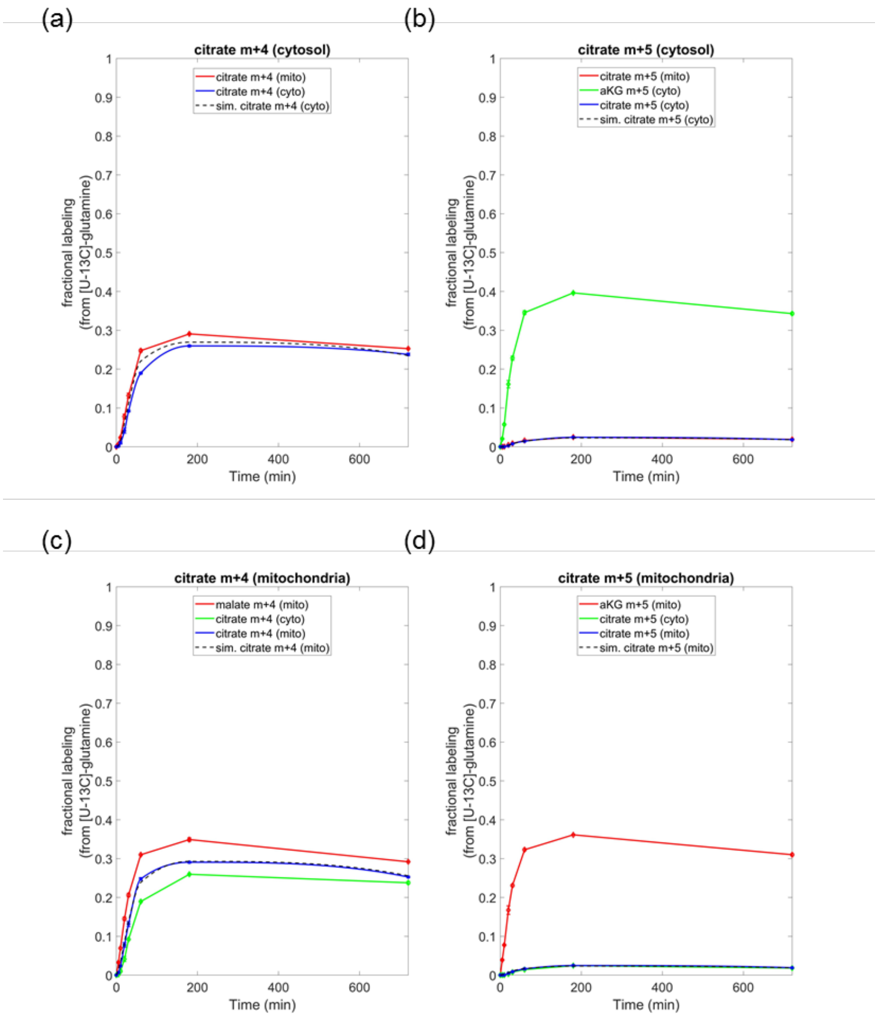

Supplementary Fig. 6: Model simulation of metabolite isotopic labeling kinetics in mitochondria and cytosol in SDH-WT cells. Measured labeling kinetics of cytosolic citrate m+4 (a) and m+5 (b), and mitochondrial citrate m+4 (c) and m+5 (d) after switching cells to [U-^{13}C]-glutamine are in blue, while the corresponding model simulations are in black. The additional curves in each subgraph show the measured labeling kinetics of substrate metabolite pools (citrate, α-ketoglutarate, and malate in mitochondria and cytosol) producing the specific simulated mass-isotopomers of citrate. Data are mean ± SD, n = 3 biological replicates.

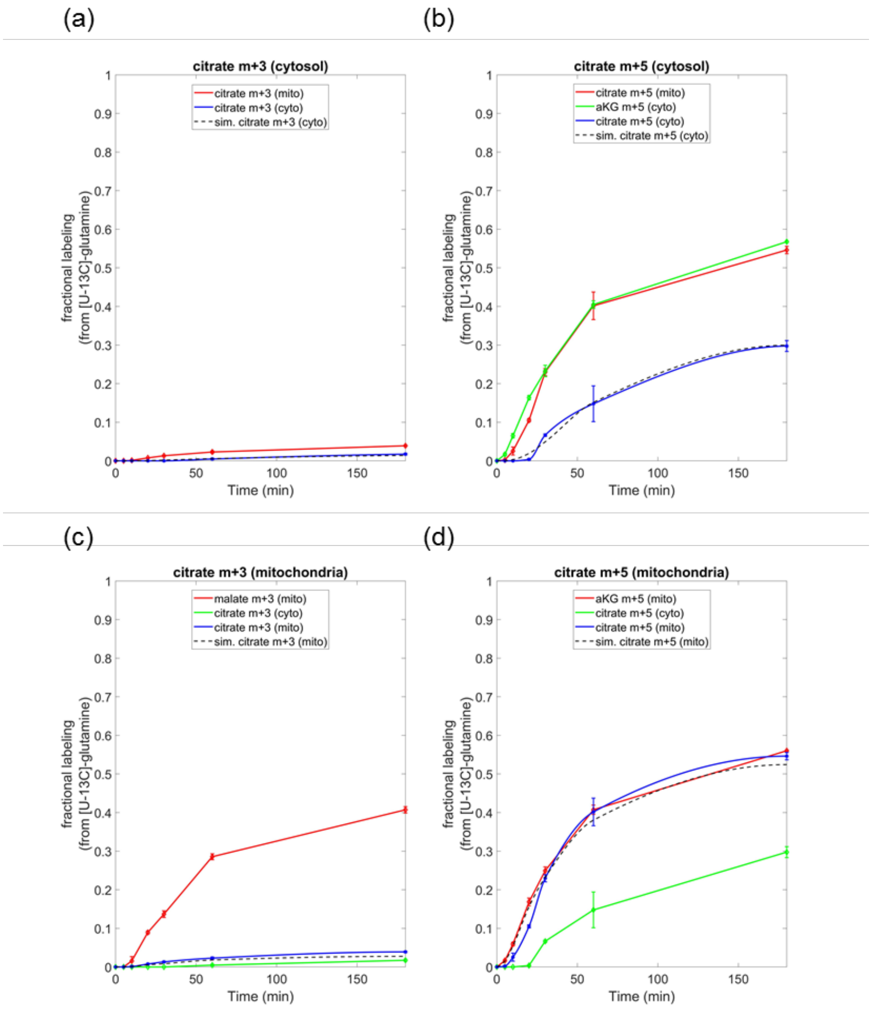

Supplementary Fig. 7: Model simulation of metabolite isotopic labeling kinetics in mitochondria and cytosol in SDH-KO cells. Measured labeling kinetics of cytosolic citrate m+4 (a) and m+5 (b), and mitochondrial citrate m+4 (c) and m+5 (d) after switching cells to [U-^{13}C]-glutamine are in blue, while the corresponding model simulations are in black. The additional curves in each subgraph show the measured labeling kinetics of substrate metabolite pools (citrate, α-ketoglutarate, and malate in mitochondria and cytosol) producing the specific simulated mass-isotopomers of citrate. Data are mean ± SD, n = 3 biological replicates.

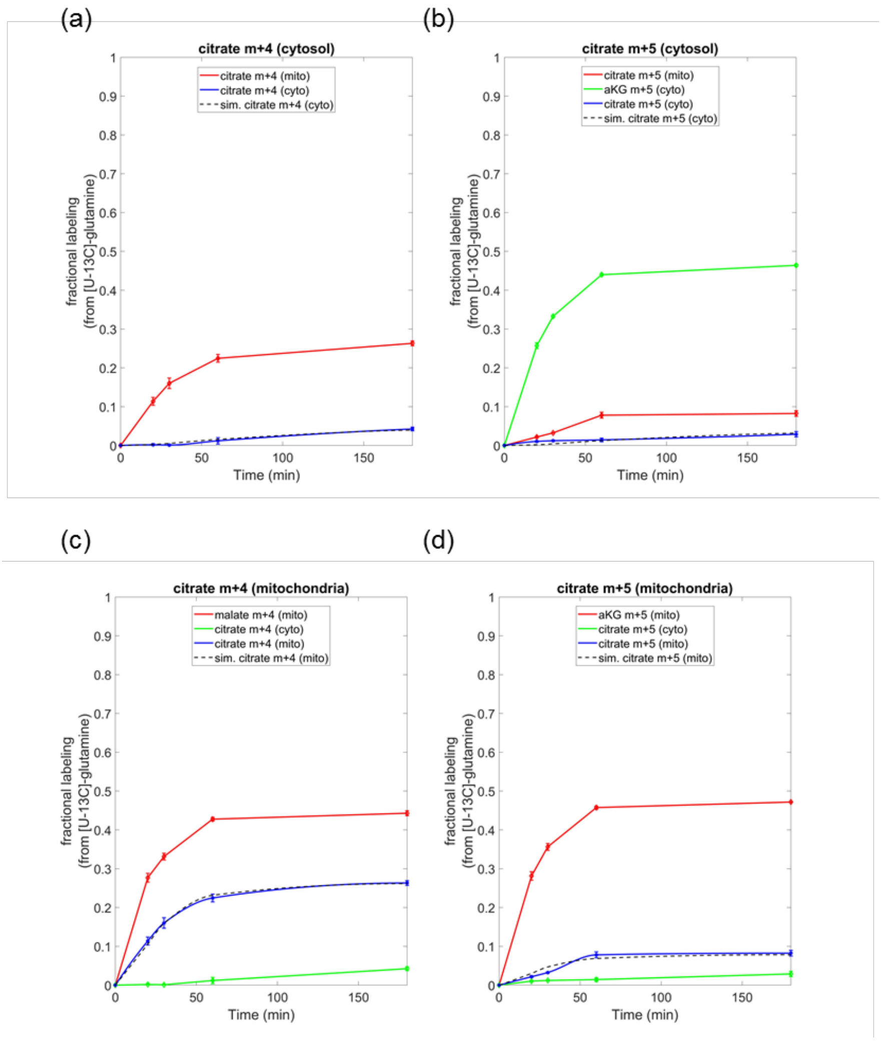

Supplementary Fig. 8: Model simulation of metabolite isotopic labeling kinetics in mitochondria and cytosol in HeLa cells grown with a physiological glutamine concentration of 0.5mM. Measured labeling kinetics of cytosolic citrate m+4 (a) and m+5 (b), and mitochondrial citrate m+4 (c) and m+5 (d) after switching cells to [U-^{13}C]-glutamine are in blue, while the corresponding model simulations are in black. The additional curves in each subgraph show the measured labeling kinetics of substrate metabolite pools (citrate, α-ketoglutarate, and malate in mitochondria and cytosol) producing the specific simulated mass-isotopomers of citrate. Data are mean ± SD, n = 3 biological replicates.

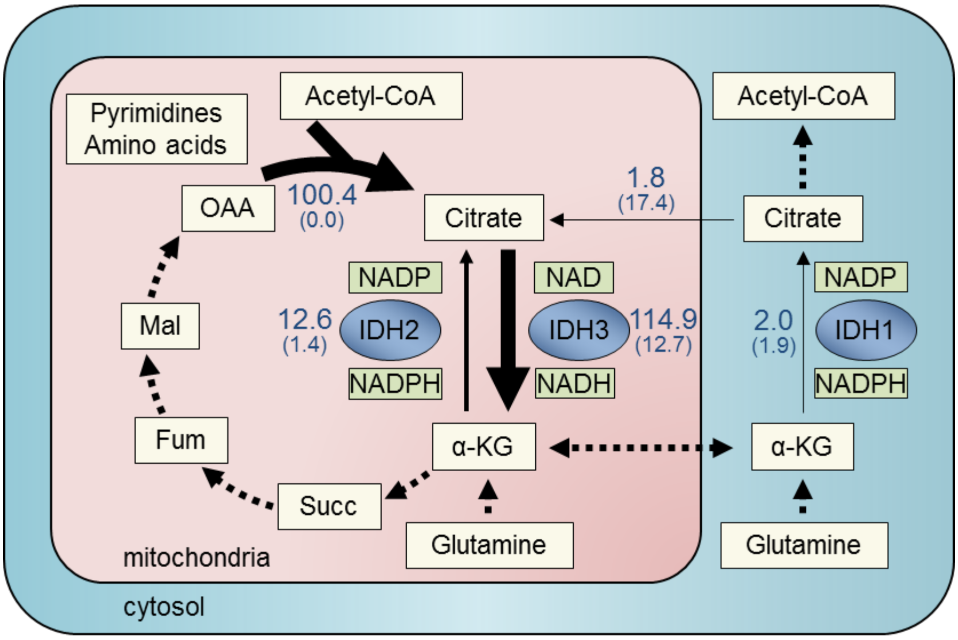

Supplementary Fig. 9: Mitochondrial and cytosolic fluxes in HeLa cells grown with a physiological glutamine concentration of 0.5 mM. Fluxes are shown in percentage from citrate synthase flux (which is 0.06 mM/h). Arrows represent the direction of net flux; numbers represent net flux in the direction of the arrow, and numbers in parenthesis represent the backward flux. Confidence intervals for estimated fluxes are shown in Supplementary Table 8.

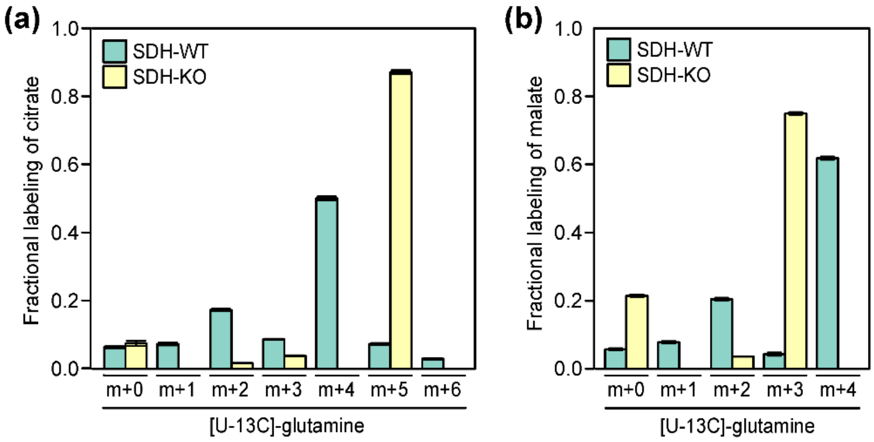

Supplementary Fig. 10: Isotopologue labeling profile of citrate (a) and malate (b) in SDH-WT (green) and SDH-KO cells (yellow) cultured for 24 h in the presence of [U-^{13}C]-glutamine.

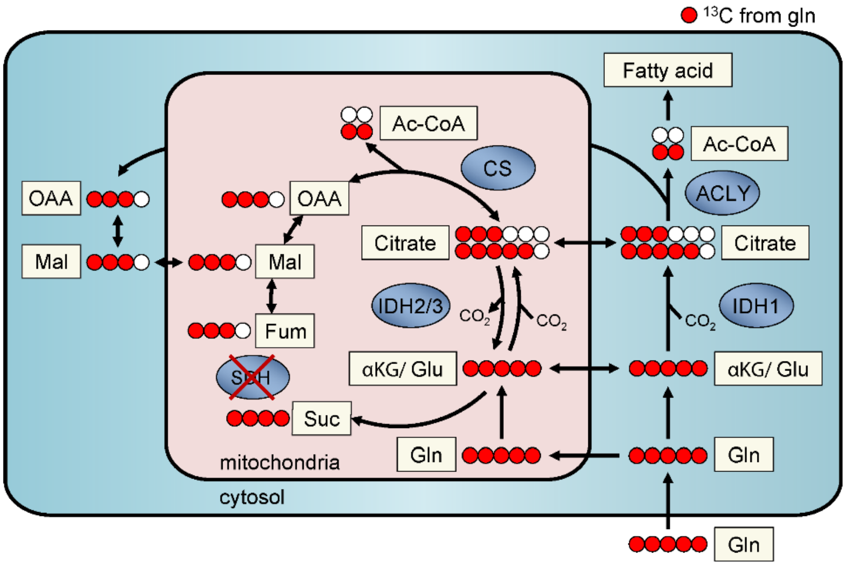

Supplementary Fig. 11: Carbon atom mapping of metabolites involved in the TCA cycle and associated reactions involving acetyl-CoA and fatty acid metabolism in SDH-deficient cells.

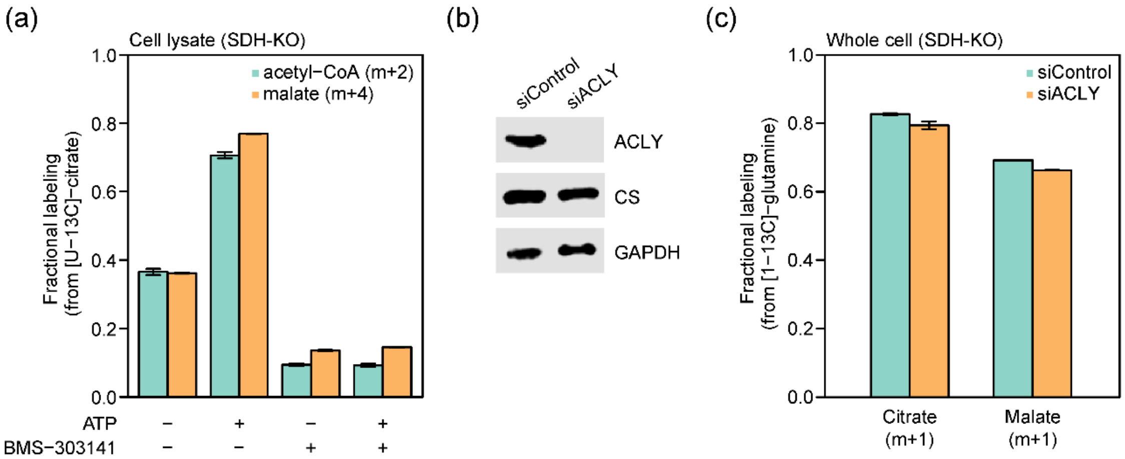

Supplementary Fig. 12: SDH-deficient mitochondria catalyze citrate cleavage via reverse CS activity. (a) Formation of acetyl-CoA (m+2) and malate (m+4) from [U-^{13}C]-citrate cleavage catalyzed by cell extracts of SDH-deficient cells. We observed ~36% of acetyl-CoA and malate being labeled in their m+2 and m+4 form, respectively, indicating citrate cleavage flux in the SDH-KO lysate. To test the activity of reverse CS specifically, we added 1 mM of the ACLY inhibitor BMB-303141. Note that, the IC50 of BMS-303141 is 8 µM; 20 µM was previously shown to completely inhibit ACLY activity2, while here we utilized a 50-times higher concentration to assure complete inhibition. We found that, while the fractional labeling of acetyl-CoA and malate dropped upon ACLY inhibition, both acetyl-CoA m+2 and malate m+4 were produced – testifying for reverse CS flux. Adding ATP while treating with the inhibitor did not increase the abundance of acetyl-CoA m+2 and malate m+4, further testifying that the high concentration of the inhibitor indeed provided complete inhibition of ACLY. (b) Immunoblots of proteins in SDH-KO cells transfected with siACLY or siControl. (c) Fractional labeling of citrate (m+1) and malate (m+1) from [1-^{13}C]-glutamine in SDH-KO cells transfected with siACLY or siControl. Feeding cells with [1-^{13}C]-glutamine for 24hrs, fractional labeling of citrate m+1 and malate m+1 remained unaffected in SDH-KO cells with or without ACLY knockdown, suggesting that ACLY contribution to citrate cleavage is insubstantial in these cells.

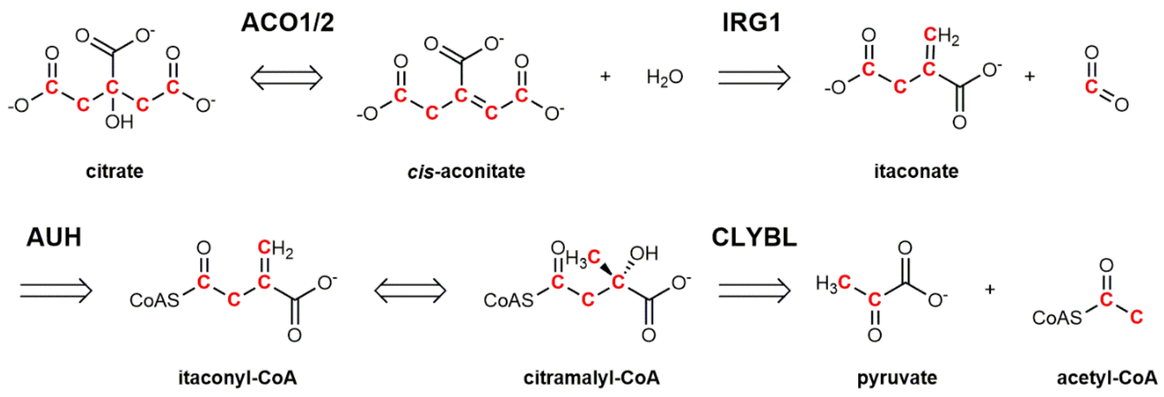

Supplementary Fig. 13: Isotope tracing of [U-^{13}C]-glutamine from reductively synthesized citrate m+5 to pyruvate m+2.

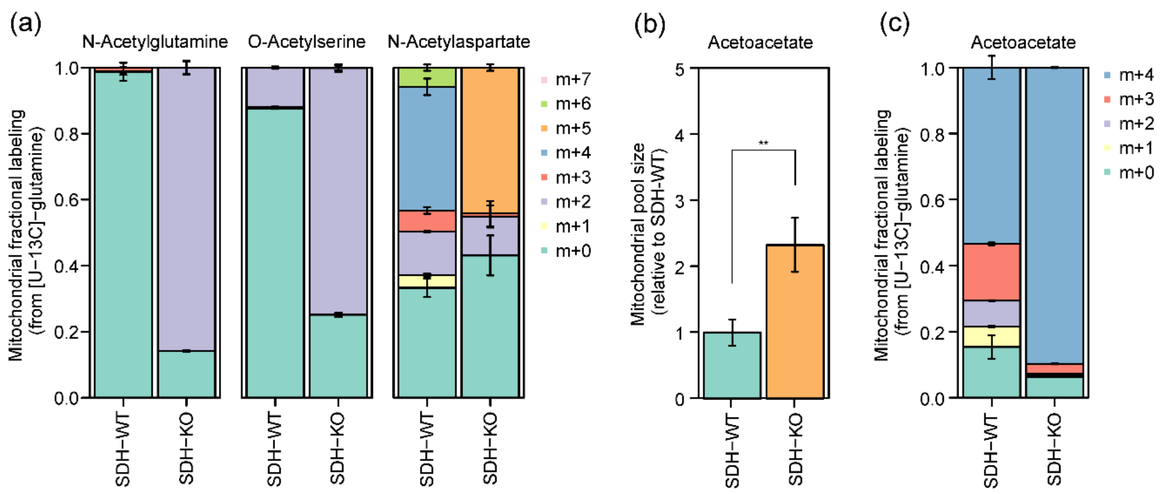

Supplementary Fig. 14: Mitochondrial acetyl-CoA from citrate cleavage participate in amino acid metabolism and ketogenesis. (a) Fractional labeling of N-acetylglutamine, O-acetylserine, and N-acetylaspartate in mitochondria. (b) Acetoacetate pool size in SDH-WT and KO mitochondria (**P < 0.01 by two-sample t-test). (c) Fractional labeling of acetoacetate in mitochondria.

Fig. 1b

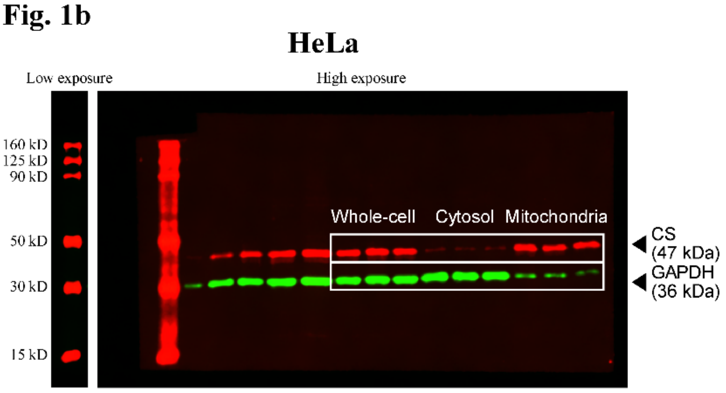

Fig. 3i

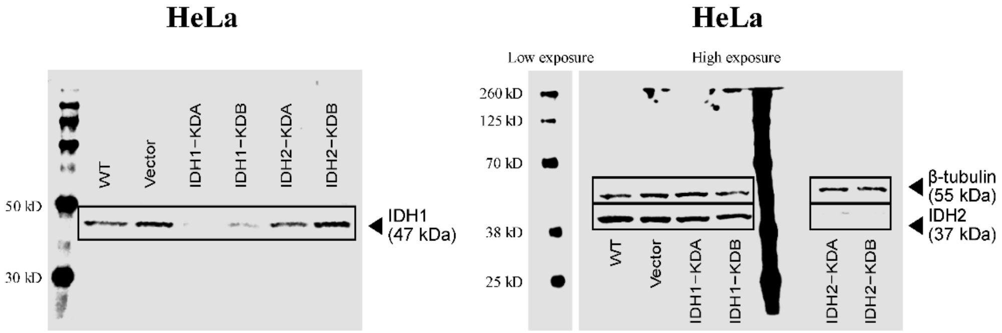

Fig. 5f

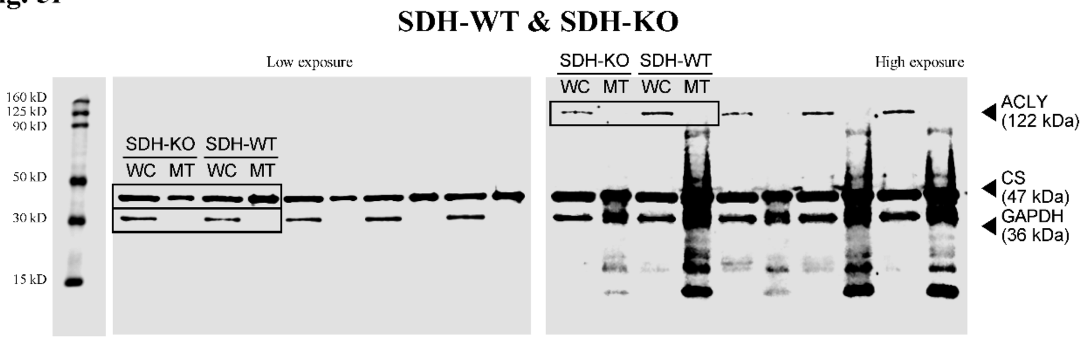

Supplementary Fig. 15. Uncropped western blot gels.
