# Supplementary material for: Spatial-fluxomics provides a subcellular-compartmentalized view of reductive glutamine metabolism in cancer cells
Source: Nat Commun. 2019 Mar 22;10:1351. doi: 10.1038/s41467-019-09352-1 (PMC6430770; doi:10.1038/s41467-019-09352-1)
Supplement: Supplementary file 3 — Description of Additional Supplementary Files [file 41467_2019_9352_MOESM3_ESM.pdf]

Description of Additional Supplementary Files

File Name: Supplementary Data 1
Description: Relative metabolite pool sizes in mitochondria and cytosol.

File Name: Supplementary Data 2
Description: Absolute metabolite pool sizes in mitochondria and cytosol.

File Name: Supplementary Data 3
Description: Mass-isotopomer distribution of metabolites in mitochondrial and cytosolic fraction under normoxia.

File Name: Supplementary Data 4
Description: Mass-isotopomer distribution of metabolites in mitochondrial and cytosolic fraction under hypoxia.

File Name: Supplementary Data 5
Description: Mass-isotopomer distribution of metabolites in mitochondrial and cytosolic fraction of SDH-WT cells.

File Name: Supplementary Data 6
Description: Mass-isotopomer distribution of metabolites in mitochondrial and cytosolic fraction of SDH-KO cells.

File Name: Supplementary Data 7
Description: Mass-isotopomer distributions of metabolites in mitochondrial and cytosolic fraction given physiological concentration of glutamine (0.5 mM).

File Name: Supplementary Data 8
Description: Inferred fluxes.
